# Saving Senior Smiles: A Community Outreach Educational Program and Pilot Research Project

**DOI:** 10.1093/geroni/igab046.3060

**Published:** 2021-12-17

**Authors:** Prajakta Joshi, Kadambari Rawal

**Affiliations:** 1 Boston University Henry M. Goldman School of Dental Medicine, Boston, Massachusetts, United States; 2 BU Henry M Goldman School of Den, Boston university, Massachusetts, United States

## Abstract

Globally, poor oral health has been evidenced more frequently among older adults. Thus, it is imperative to develop strategies for improving the oral health knowledge and access to dental care amongst the older adult population. The Saving Senior Smiles (S3) pilot outreach program was launched as an oral health education and awareness program for community-dwelling older adults across senior centers in the greater Boston area (Massachusetts, USA). The outreach consisted of oral health educational seminars presented by pre- doctoral dental students from three dental schools in the Boston area. The presentations highlighted the significance of oral health, and the importance of seeking routine dental care. Pre and post-test surveys were administered to assess the participants’ utilization of oral health services and oral health knowledge. The surveys were completed by 85 older adults (Female= 58.8%) across five senior centers. Questions pertaining to utilization of dental services revealed that 78.8% of the participants had a dentist. Expectedly, the center that reported the greatest number of missing teeth (Fenway center= 70.6%) had the least number of individuals who had a dentist (58.3%). With regard to oral health knowledge, before the seminars, less than half of the participants (42.2% ) across all the senior centers were aware of the common oral conditions that affected older adults ( dry mouth, gum recession and changes in oral bacteria) and after the presentation over 60% of the participants responded correctly to these knowledge questions. Overall, these findings emphasize the value of simple community-based interventions for older adults.

